# Evaluating the Impact of Metro Interior Noise on Passenger Annoyance: An Experimental Study

**DOI:** 10.3390/ijerph19095041

**Published:** 2022-04-21

**Authors:** Meng Ma, Wenqian Ran, Jinping Wu, Minghang Li, Xiangyu Qu

**Affiliations:** 1 School of Civil Engineering, Beijing Jiaotong University, Beijing 100044, China; 18231378@bjtu.edu.cn (W.R.); 18231387@bjtu.edu.cn (J.W.); 2 Urban Rail Transit Center, China Academy of Railway Sciences Corporation Limited, Beijing 100081, China; lmh_rails@126.com; 3 Institute of Sound and Vibration Research, University of Southampton, Southampton SO17 1BJ, UK; xq2n20@soton.ac.uk

**Keywords:** metro, interior noise, noise test, annoyance rating, exposure-response relationship

## Abstract

The operation of a large-scale metro system creates problematic interior noise; the impact of this noise on passengers and drivers is a subject of increasing concern. To investigate the quantitative relationship between metro interior noise and passengers’ annoyance, this study analyzed questionnaires on passenger annoyance completed by 118 volunteers. The feedback from the questionnaire concerned eleven metro lines in Beijing. To test the interior noise levels, the volunteers were divided into two groups: A and B. The volunteers in group A took the same metro train as the testers, whereas those in group B took different trains. A total of 2080 noise annoyance samples from metro tunnel sections were collected and analyzed. Finally, the exposure-response relationship between interior noise and passenger annoyance was obtained by fitting these data with a logistic function. The results indicated that there was a significant positive correlation between the average subjective annoyance and the averaged equivalent sound pressure level. The fitting result was better for group A than for group B. For the mixed samples of two groups, the fitting result was greatly affected by the contribution of group A. To provide an acoustically comfortable environment, metro interior noise should not exceed 84–85 dB(A).

## 1. Introduction

Traffic-induced vibration and noise pollution are major contributors to environmental complaints in large cities; they also have negative impacts on human health and well-being. The health effects of exposure to noise include the sleep disorders with awakenings [1], learning impairment [2,3,4], hypertension ischemic heart disease [5,6], diastolic blood pressure [7], reduction in working performance [8,9], annoyance [10,11]. With the continuous construction of urban rail transit systems and the densification of metro networks, the problem of train-induced vibration and noise has gained increasing attention in recent years [12,13,14,15,16].

One type of this problem comprises train-induced building vibration and associate structure-borne noise, which have adverse effects on the sleep patterns of residents near railway lines. Elmenhorst et al. [17] compared the effects of road, railway, and aircraft noise on sleep; they indicated that railway noise may have the most disturbing properties on sleep. The exposure-response relationships between vibration and noise and annoyance have been investigated to evaluate the impacts of railway vibration and noise impacts on human beings [12,13,14,15,16,17,18,19,20,21]. To mitigate the impact of railway vibration on residents, measures can be taken at the source [22,23,24,25,26], along the propagation path [27,28,29], and at the receivers [30,31,32]. Similarly, the impacts of railway noise can be controlled by introducing Technical Specification for Interoperability (TSI) limit values, reducing roughness, increasing wheel damping, building noise barriers, or installing insulated windows [33,34].

Another type of the problem is the interior noise, which has the adverse effects on the train drivers and passengers [35]. Investigations of railway passengers show that the sound comfort and sound environment inside of trains are important factors regarding train comfort [33]. Furthermore, the long-term exposure to train interior noise can affect people’s moods and even their health. According to a statement issued by the World Health Organization (WHO), exposure to a noise environment over 85 dB(A) for more than 45 min can cause noise-induced hearing loss [36]. Interior noise is composed of air-borne noise, which is generated by external sources, and structural noise, which mainly includes the wheel/rail rolling effect, sleeper passing frequency excitation, and aerodynamic effects. Thompson [37] indicated that wheel/rail roughness is one of the main sources of interior noise excitation, especially when the rail is corrugated. The running speeds of metro trains are usually between 30 and 80 km/h. At this speed, the wheel/rail noise becomes a problem that cannot be ignored.

To investigate and evaluate metro interior noise, East and Hardy [38] analyzed the mechanisms by which noises generated by various noise sources are transmitted through air-borne and structure-borne paths into the trains. Forssén et al. [39] proposed a statistical energy analysis model to predict the interior sound fields of railway vehicles. Han et al. [40] tested and analyzed the influence of train speed on interior noise. Li et al. [41] tested metro interior noise and vibration at different train speeds for rail fasteners with two different values of stiffness. Yan et al. [42] performed an experimental study of the interior noise and vibration of the Guangzhou metro line 14. This study indicated that interior sound pressure levels (SPLs) were affected by train speed, but that the two dominant frequencies (125–250 and 400–1000 Hz) were almost independent of train speed. Vibration reduction tracks can be employed to control train-induced environmental vibrations. However, this may cause higher interior noise. A test conducted by Wang et al. [43] indicated that interior noise and carriage floor vibrations on a floating slab track were obviously higher than those on a regular slab track.

Measures such as rail grinding and rail dampers are suggested to control interior noise. Zhao et al. [44] found that an effect (3 dB(A)) could be obtained by grinding corrugated rails, and that an additional effect (8 dB(A)) could be obtained by using rail dampers. Han et al. [40] also analyzed the influence of rail corrugation on metro interior noise; they proposed a rail grinding control method based on noise limits.

A-weighted SPL is commonly used to evaluate interior noise. However, this approach may underestimate the impacts of the low-frequency components of interior noise on passengers, especially regarding their interior acoustic comfort [45,46]. Research shows that it is impossible to improve an interior acoustic environment only by reducing A-weighted SPL. Passengers’ subjective perceptions should be taken into account when assessing acceptable train interior noise [47,48].

The above review shows that the characteristic analyses, test and evaluation and control measures of interior noise are widely studied. Although its negative effect on passengers has been recognized and widely investigated, few studies were conducted into the exposure-response relationship between metro interior noise and passenger annoyance. To address this problem, in the present study, passenger sound comfort was investigated with the help of 118 volunteers. Feedback was obtained from a questionnaire that was based on eleven metro lines in Beijing. Meanwhile, interior noise levels were also measured. Finally, the exposure-response relationship and corresponding mathematical expression were obtained by fitting a logistic function to the experimental samples.

## 2. Methods

### 2.1. Noise Annoyance Investigation

#### 2.1.1. Data Collection Design

To obtain the exposure-response relationship between metro interior noise and passenger annoyance, both noise measurement and annoyance were measured. As individuals’ subjective perceptions to noise can vary greatly, evaluating noise annoyance results from a single passenger is of little reference value. Rather, it is more valuable to measure the percentage of passengers annoyed by interior noise.

When analyzing the vibration and noise annoyance of residents near railway lines, statistics based on data from self-reported annoyance or questionnaire surveys can be used. However, this method is not applicable to analyzing metro interior noise annoyance. On the one hand, when metro trains run through different running tunnels and station sections, the interior noise is greatly affected by the train’s speed, the metro routine radius, the track type, and the rail wear state. Passengers cannot accurately recall their subjective experience of being annoyed by interior noises in the past, especially regarding their specific annoyance feelings for each running tunnel section. On the other hand, most passengers are not willing to cooperate with questionnaire surveys when traveling on a metro. Due to the generally short commuting times of passengers using metros, they have usually already arrived at their destination before they can be told the purpose of the investigation and the methods and rules for filling in the questionnaire. Moreover, the different destinations of each passenger greatly increase the difficulty of issuing and withdrawing questionnaires.

To solve the problems above, 118 volunteers were employed to perform subjective annoyance assessments. The purpose and significance of the study, as well as the rules for completing the questionnaire, were clearly explained to each volunteer in advance. When they took the metro to work, back home, shopping, and traveling, they consciously paid attention to interior noise. They gave subjective scores for each running tunnel section and answered corresponding questions in the questionnaire.

Due to the limitations of the numbers and time arrangements of the noise testers, as well as the time arrangements of the volunteers taking the subway, it was difficult to arrange for one tester to follow every volunteer taking the metro. Therefore, the volunteers were divided into two groups: A and B. The volunteers of group A traveled the metro with the testers. That is, the measured interior noise level was highly correlated with the volunteers’ annoyance feedback, as they were in the same train car. Testers did not follow the volunteers of group B when they took the metro. In the questionnaires, volunteers provided detailed information of the metro lines they took and the stations and sections through which they traveled. After obtaining this information, testers then tested the interior noise in the metro lines where the volunteers had traveled. As the volunteers and testers likely did not travel in the same train cars in group B, the correlation between subjective annoyance feedback and the measured noise level in this group was much lower than that in group A. The sample validity of group B was also tested in this study.

#### 2.1.2. Questionnaire Design

The noise annoyance questionnaire included three parts: passenger’s basic information, subjective rating of noise annoyance (score of *ψ*), and a short description on perception; details are listed in Table 1.

For the item ‘basic information’, volunteers were asked to provide their sex, age, current occupation, and the metro line they took.

For the item ‘Subjective rating of noise annoyance’, volunteers were instructed to give a score on an 11-point categorical scale (from zero to ten) for each section between two stations. The leaving from station and approaching station of each section were also filled by the volunteers. In the fourth column of this part ‘subjective rating’, they were told that scores *ψ* ≥ 8 represented a highly annoyed perception; scores *ψ* ≥ 5 represented an annoyed perception; scores *ψ* ≥ 3 represented a slightly annoyed perception; and scores *ψ* < 3 represented no annoyance. This rating rule is suggested by some standards and was used in similar studies, e.g., ISO/TS 15666 [49], DELTA [50], and Zou et al. [51].

For the item ‘Perception description’, volunteers were asked to provide some subjective description of the main effects of the interior noise if they rated a high subjective score.

#### 2.1.3. Participants’ Characteristics

**Figure 1:**
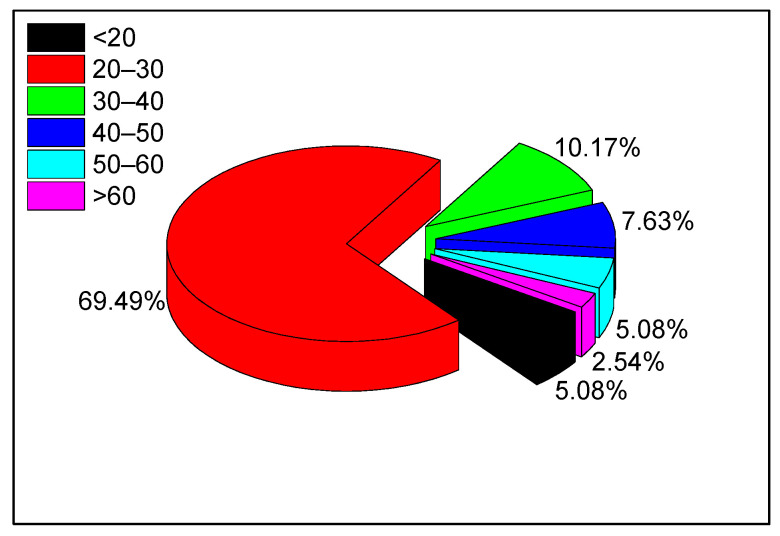
Volunteers’ age distribution.

A total of 118 volunteers were involved in the noise annoyance survey, with 61% (*n* = 72) males and 39% (*n* = 46) females; their ages ranged from 18 to 68 years old. Most volunteers were university students and office workers, and were between 20 and 30 years old. These volunteers commuted daily using the metro, and these groups represent the main sources of metro passengers. The volunteers’ age distribution is shown in Figure 1. The volunteers were split equally between groups A and B.

### 2.2. Interior Noise Measurement

Two types of noise measurement equipment were used. One was an integrated circuits piezoelectric sound pressure sensor. It has a measurement range between 20 and 146 dB(A). In the frequency range between 20 and 20,000 Hz, its technical indices can meet the requirement of Class 1 according to the standards of IEC 61672-1 [52]. The second was a multi-function portable sound level meter, which has a measurement range between 20 and 142 dB(A). In the frequency range between 10 and 20,000 Hz, its technical indices can meet the requirement of Class 1 according to the standards of IEC 61672-1 [52]. Before the test, both types of equipment were calibrated.

**Figure 2:**
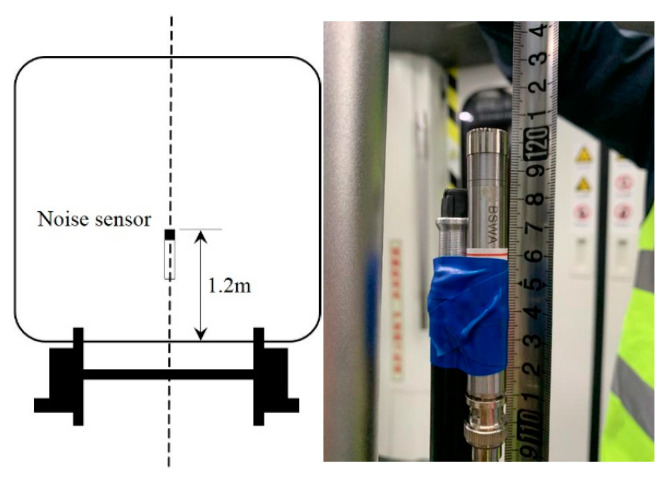
Noise sensor location.

Considering the passengers’ seated positions, the microphones were located at a height of 1.2 m above the vehicle floor, following the suggestion of the national standard GB 14892 [53] and the international standard ISO 3381 [54], as illustrated in Figure 2. As the microphones were located at the center in the vehicle, this placement ensured them to be at least 1 m from reflecting surfaces.

In the interior noise test, the discontinuous sampling mode was used. Each sampling included the whole process from the train leaving the previous station to it reaching the next station. Supposing that the train running time between two stations was *T* = *t*_2_ − *t*_1_, the starting time, *t*_1_, corresponded to the moment when the doors were completely closed, and the train was leaving the previous station. Furthermore, the ending time, *t*_2_, corresponded to the moment when the train stopped completely but the door had not yet opened after reaching the next station.

To evaluate the interior noise, the A-weighted equivalent SPL was used as a main index.

## 3. Results

### 3.1. Subjective Evaluation of Noise Annoyance

**Figure 3:**
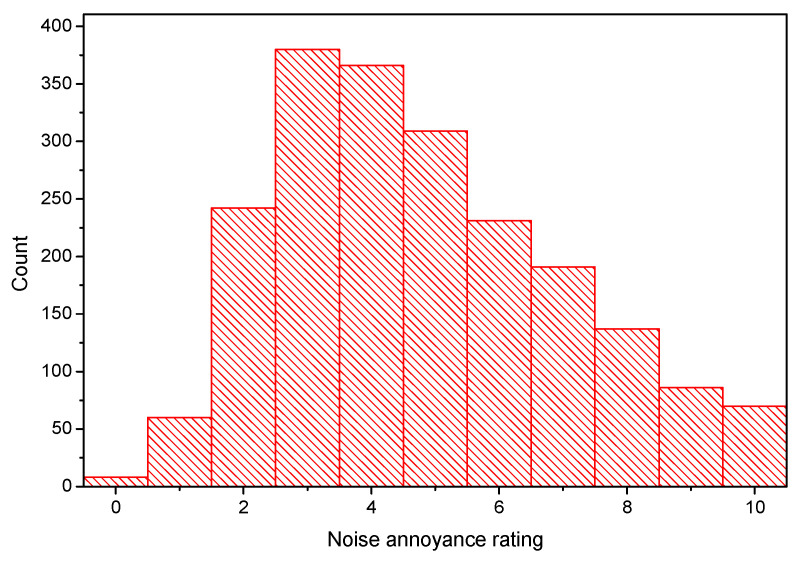
Statistics of subjective annoyance rating samples.

**Figure 4:**
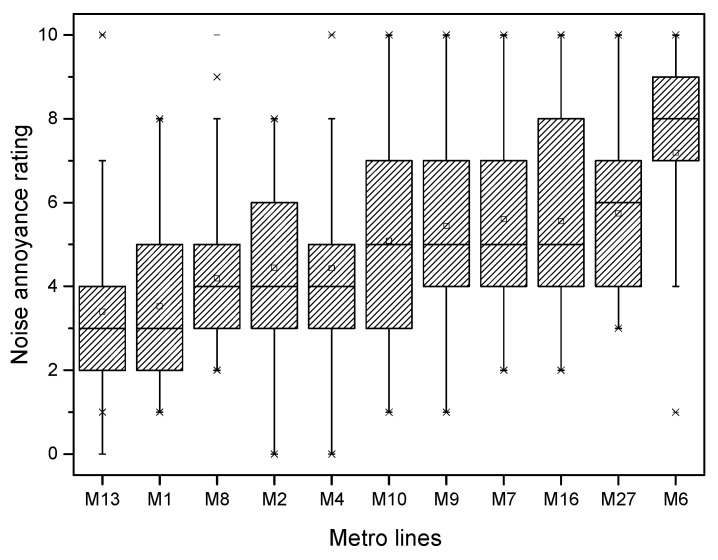
Subjective annoyance ratings of interior noises for different metro lines.

The numbers of annoyance rating samples from the two groups were 1030 and 1050, respectively. A total of 2080 annoyance samples were obtained, and each volunteer took an average of 17.6 running tunnel sections. Figure 3 illustrates the statistical results of the noise subjective annoyance rating for all 2080 samples, including both groups A and B. Highly annoyed (*ψ* ≥ 8), annoyed (*ψ* ≥ 5), and slightly annoyed (*ψ* ≥ 3) scores accounted for 14.1%, 49.2%, and 85.1% of all data, respectively. The 2080 subjective rating samples were from eleven metro lines, i.e., metro lines M1 (including its east extension), M2, M4, M6, M7, M8, M9, M10, M13, M16, and M27. Figure 4 shows the statistics of the subjective scores of volunteers for each metro line. M6 was clearly rated as the ‘noisiest’ metro line. Due to the high maximum running train speed (100 km/h) and the serious rail corrugation, the passengers were used to complaining about the interior noise of M6; this issue is also reported on by news media [55]. The rating results from the volunteers also objectively reflected this fact. M13 was rated as the ‘quietest’ metro line. As M13 does not pass through urban areas, it travels only on ground level and elevated lines, without going underground. In addition, this line employs the ballast track. These may be the reasons for its low noise annoyance to passengers.

**Figure 5:**
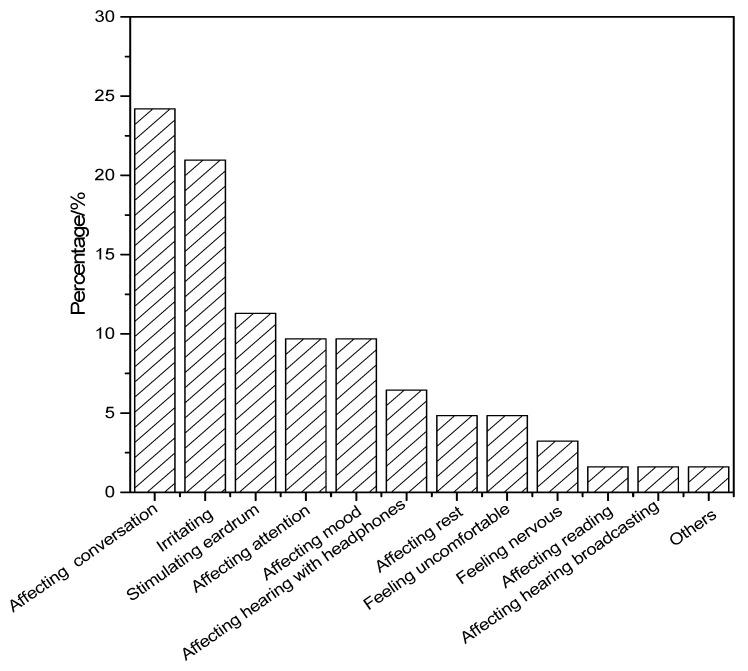
Main noise effects on passengers.

The main effects of interior noise on volunteer passengers were analyzed based on the third part of the questionnaire. As illustrated in Figure 5, the impact of high annoyance noise on passengers was multifaceted; it included normal communication, psychological, and physiological aspects. The top three effects were *affecting conversation*, *irritating*, and *simulating eardrum*.

### 3.2. Interior Noise Analysis

**Figure 6:**
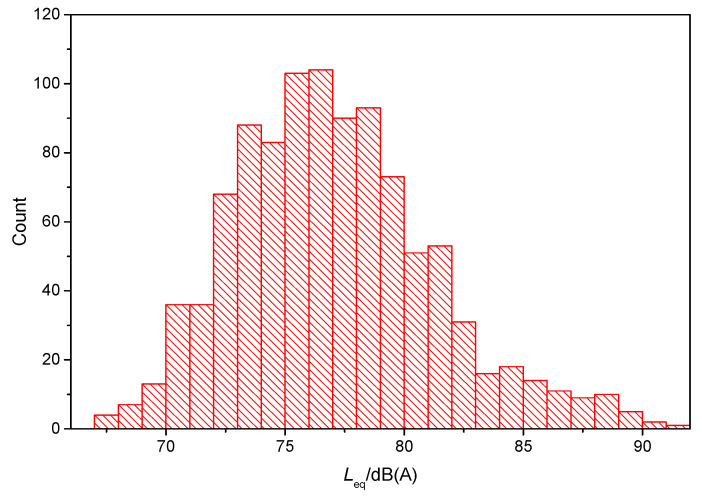
Statistics of equivalent SPL.

Figure 6 illustrates statistical results of equivalent SPLs for all 1019 noise test samples, which correspond to the 2080 annoyance scores. In group A, several volunteers and the tester were in the same metro vehicle, whereas in group B, different volunteers traveled through the same sections. Therefore, one noise test sample from group A, or an averaged noise test sample from group B, corresponded to multiple annoyance samples. Accordingly, in the interior noise analysis, the sample number of equivalent SPLs was smaller than that of the annoyance score. Figure 6 reveals that 18.8% and 3.6% of measurements were greater than or equal to 80 dB(A) and 85 dB(A), respectively.

**Figure 7:**
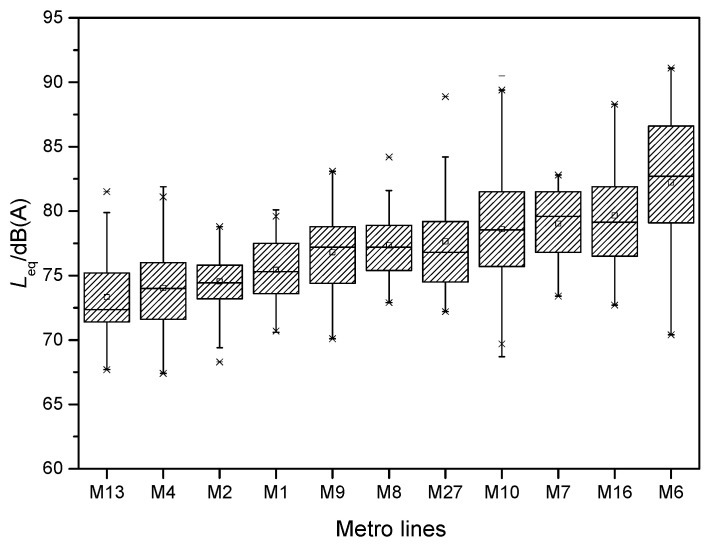
Equivalent SPL of interior noises for different metro lines.

The 1019 noise test samples were also from the eleven metro lines mentioned above. Figure 7 shows the statistics of the equivalent SPL for each metro line. The interior noise of M6 was clearly the largest, whereas that of M13 was the smallest. These lines also exhibited the largest and smallest subjective scores, as shown in Figure 4. For the other metro lines, however, the relationship between the subjective score and the test noise did not correspond completely.

### 3.3. Measurement Error Analysis

#### 3.3.1. Noise Difference between Sensor Locations inside a Vehicle

The measured SPL inside a vehicle may vary considerably depending on the location. However, even if the tester and the volunteer were in the same car (i.e., in group A), the microphones could not be placed immediately adjacent to the ears of the volunteers. That is, there was a difference between the noises that the volunteers heard and those that the microphones collected. This error was unavoidable in this study. Therefore, it was necessary to analyze the noise differences at different positions within a vehicle.

**Figure 8:**
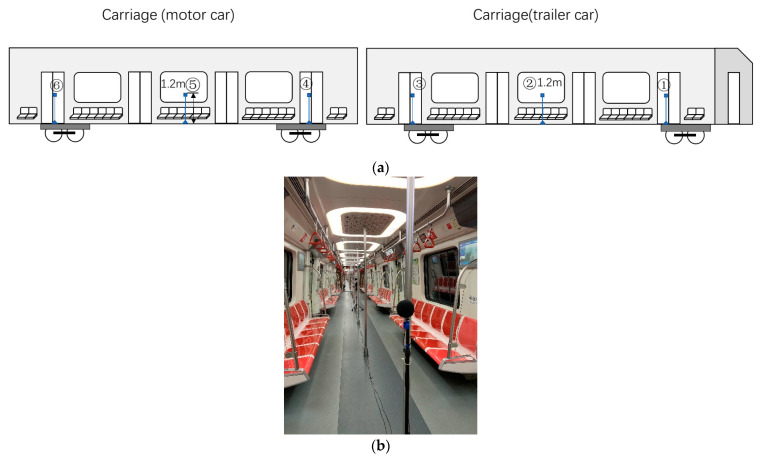
Microphones in the error analysis of sensor location. (**a**) Microphone arrangement; (**b**) Test picture.

To avoid the effect of passenger conversations on the test results, this error analysis was arranged in a special metro train without passengers. Six microphones were located in two adjacent vehicles (a motor car and a trailer). The microphones were arranged in the front, middle, and rear of each vehicle (Figure 8).

**Figure 9:**
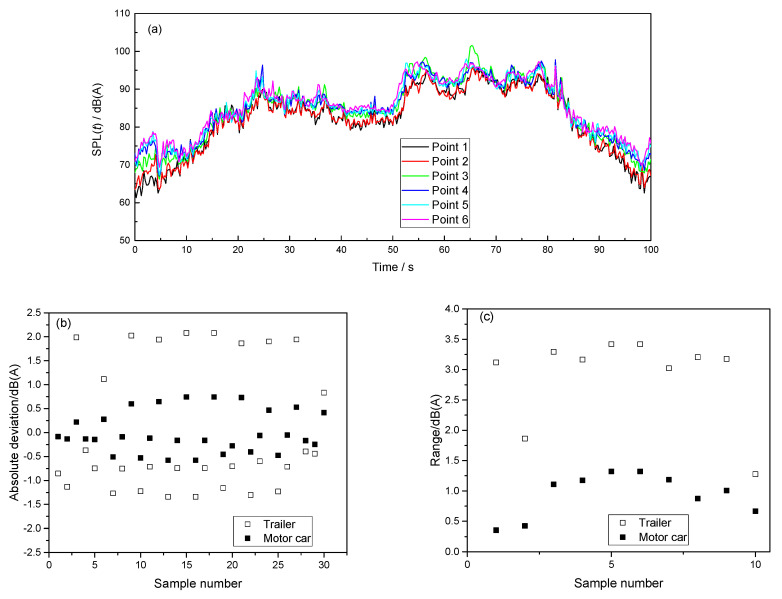
Measurement error analysis: (**a**) a typical running SPL(*t*) of a metro section between two stations, (**b**) the absolute deviations of the ten tested metro sections, and (**c**) the ranges of the ten tested metro sections.

Figure 9 illustrates the measurement error between the sensor locations inside the vehicles. Generally, the test errors in the trailer were larger than those in the motor car. The largest absolute deviation was approximately 2 dB(A), and the largest range was approximately 3.5 dB(A). Other studies also measured differences in interior metro noise at different locations in a vehicle. Nong et al. [56] arranged eight microphones in a metro vehicle and on the section above the bogie. Their test results showed that the noise near the door and window was approximately 1.5 dB(A) larger than that at the middle position at the same height, and that the noise range was between 0.8 and 4.3 dB(A) within different test conditions. Liu et al. [57] arranged five microphones along the length of metro vehicles. They found that when trains ran at different speeds, the noise range at different positions in a car was between 0.8 and 5.4 dB(A), and with an average of 3.3 dB(A). From the test results in this study and those conducted previously, the highest measurement errors due to the microphone location were determined to be between approximately 3.5 and 5.4 dB(A).

#### 3.3.2. Noise Difference between Different Vehicles or Trains

**Figure 10:**
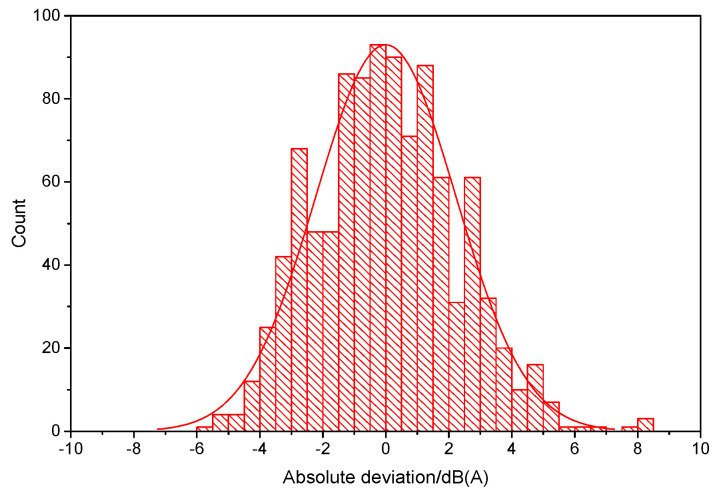
Absolute deviation of tested interior noise in different vehicles running in the same metro section.

In group B, the vehicles’ noise tests were conducted differently from the vehicles taken by passengers. Therefore, it was necessary to learn the noise difference inside different vehicles when they were running through the same running tunnel section. Then, the interior noises were repeatedly tested in 197 tunnel sections; a total of 1010 samples were collected. Figure 10 illustrates the absolute deviations of repeatedly tested samples. The statistical results showed a close to normal distribution, with a mean value of zero. Ninety-five percent of the samples were within ±4.25 dB(A) deviation. The maximum range reached approximately 8.5 dB(A). This type of test error was much larger than the error observed at different positions in the same vehicle. This reflects that the differences between vehicles, especially the differences in the wheel wear, were huge. Accordingly, this difference was considered in the subsequent analysis of group B.

### 3.4. Exposure-Response Relationship

**Figure 11:**
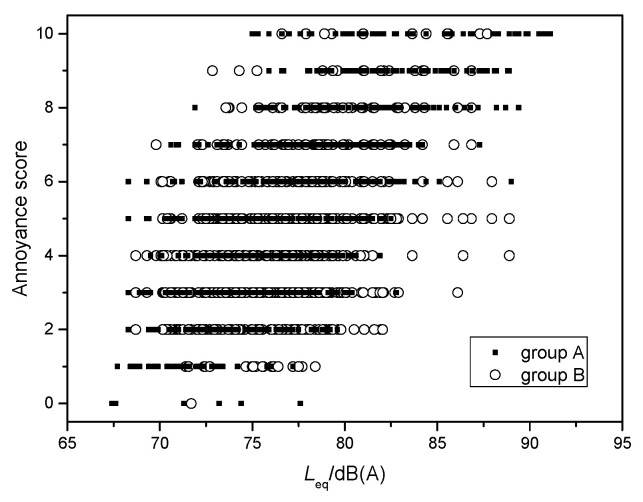
Comparison of all equivalent SPLs and annoyance scores.

The dose-response curve is commonly used to describe the relationship between noise exposure and annoyance. This curve can be obtained by fitting the samples of noise tests and annoyance rating scores. Figure 11 illustrates all equivalent SPL data and annoyance scores, including groups A and B. The data were very discrete. The same SPL values often corresponded to different subjective annoyance scores; the score difference approached ten points in some cases. For the same annoyance score, however, the maximum difference in test SPL values exceeded 20 dB(A). On the one hand, the SPL differences corresponding to one annoyance score were far greater than the differences of noise distribution in the vehicle, and greater than the interior noise difference between vehicles. On the other hand, the very discrete samples also exhibited that a simple subjective annoyance score had no reference value. Thus, it was not possible to evaluate interior noise based on one person’s annoyance score. However, careful observations still revealed that with increasing SPL, the annoyance score also increased. For example, when a volunteer’s rated score was one, the corresponding SPL range was between 67.7 and 78.4 dB(A), whereas when a volunteer’s rated score was ten, the corresponding SPL range was between 75 and 91.1 dB(A), which is significantly higher than the former.

**Figure 12:**
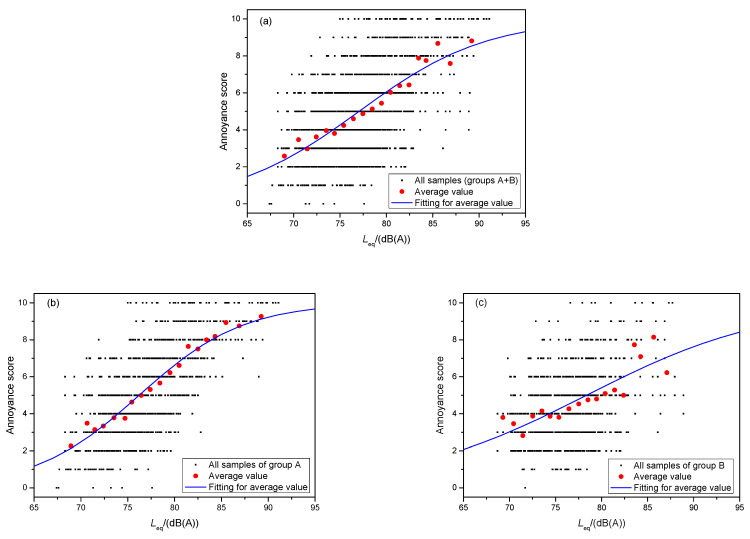
Fitting for the average annoyance score and equivalent SPL values, (**a**) for all samples of groups A and B, (**b**) for samples of group A, and (**c**) for samples of group B.

To observe and describe the relationship between annoyance and SPL better, samples were averaged and fitted with the logistic function. First, the range of SPL values was divided into 19 sections with an interval of 1 dB(A). Both the annoyance scores and SPL values in each section were averaged, as illustrated in Figure 12 using red solid dots. In Figure 12, analyses of the samples for the two groups, group A only, and group B only are shown.

A logistic function is suggested as a method to describe average scores [50,51]; it is defined as:

(1) $$\psi =\frac{u}{1+e^{-k\left(L_{\mathrm{eq}}-L_{c}\right)}}$$

where *ψ* is the annoyance response or the subjective rating score; *u* is the upper limit of *ψ* (ten in this study); *k* is the slope of the inverse logistic function, which is a constant to be fitted; *L*_eq_ is the test equivalent SPL; and *L*_c_ is the value of *L*_eq_ for a 50% annoyance response, which is the mean value of the normal distribution. By fitting the average values using Equation (3), the unknown parameters to be determined were obtained; they are listed in Table 2.

In Table 2, *adj*-*R*^2^ is the adjusted coefficient of determination, which was used to evaluate the goodness of fit. The coefficient of determination, *R*^2^, is defined as:

(2) $$R^{2}=1-\frac{RSS}{TSS}=1-\frac{\sum_{i=1}^{n}{\left(y_{i}-{\hat{y}}_{i}\right)}^{2}}{\sum_{i=1}^{n}{\left(y_{i}-\bar{y}\right)}^{2}}$$

where *RSS* is the residual sum of squares; *TSS* is the total sum of squares; $y_{i}$ is the measured value; ${\hat{y}}_{i}$ is the estimated value; and $\bar{y}$ is the mean of the measured value. As the coefficient of determination is affected by the number of fitting data points, the *R*^2^ value can be improved by increasing the number of samples. To eliminate this effect, the adjusted coefficient of determination, *adj*-*R*^2^, was introduced; this is defined as:

(3) $$adj-R^{2}=1-\frac{\left(1-R^{2}\right)\left(n-1\right)}{n-p-1}$$

where *n* is the sample number, and *p* is the number of features.

The closer the values of *R*^2^ and *adj*-*R*^2^ are to one, the better the fitting effect is. The fitting result for group B only was unacceptable; it had an *adj*-*R*^2^ value of only 0.7523. This occurred because the subjective annoyance scoring and noise testing were carried out separately. The fitting result for group A was better (*adj*-*R*^2^ = 0.9789). It is interesting that the *adj*-*R*^2^ value for all samples of groups A and B was 0.9512, which was also acceptable. This reflects that the sample quality of group A was very good. In the mixed samples, the dominant sample from group A compensated for the deficiency of group B to a certain extent. When 50% of the samples from the two groups were mixed to fit, the effect fitting the group B samples was greatly improved.

**Figure 13:**
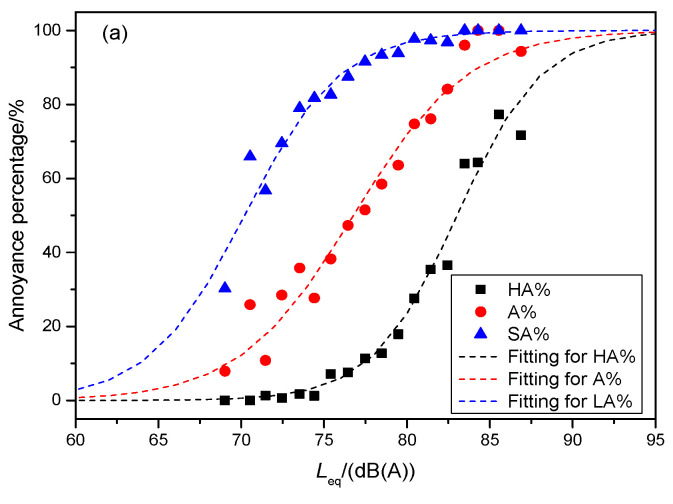

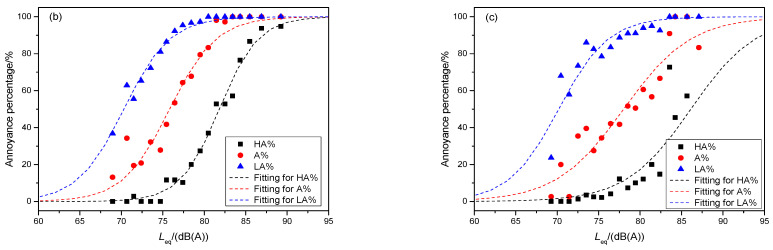
Fittings for the average values of annoyance percentages and equivalent SPLs: (**a**) for all samples of groups A and B, (**b**) for the samples of group A, and (**c**) for the samples of group B.

Based on analyzing all samples of the equivalent SPL and annoyance scores shown in Figure 11, the percentage of highly annoyed (HA%), annoyed (A%), and slightly annoyed (LA%) responses were calculated. The exposure-response relationships between the annoyance percentage and test noise are shown in Figure 13. These fitting curves were also plotted using a logistic function similar to Equation (3):

(4) $$\psi \%=\frac{u}{1+e^{-k\left(L_{\mathrm{eq}}-L_{c}\right)}}$$

where *ψ*% is the annoyance percentage; *u* is the upper limit of *ψ*% (here with a value of 100); and the meanings of other variables are consistent with those defined in Equation (3).

The unknown parameters were obtained by fitting the average values using Equation (4), as listed in Table 3. Analysis of the values of *adj-R*^2^ showed that the fitting evaluation result was similar to that in Table 3. The fitting result was best for the samples of group A and was worst for the samples of group B (this result was unacceptable). For the mixed samples of two groups, the fitting result was greatly affected by the contribution of group A.

## 4. Discussion

### 4.1. Annoyance Rating and Noise Test Error

From Figure 3, it can be calculated that the percentages of highly annoyed, annoyed, and slightly annoyed responses were 14.1%, 49.2%, and 85.1%, respectively. The proportion of highly annoyed responses is remarkably high; however, it should be noted that the research method in this study may have induced volunteers to give higher scores, especially for ‘annoyance’ and ‘slight annoyance’. Taking the metro with the questionnaire may have prompted volunteers to pay more attention to noise. If the interior noise increased significantly, the volunteers were likely to give a high annoyance score. This meant that volunteers paid more attention to the SPL and may have neglected that the score itself was for subjective annoyance. Furthermore, when passengers normally take the metro, they may listen to music, read, browse mobile phones, and chat with their peers, which will distract their attention from the noise itself. However, to complete the questionnaire, volunteers were asked to focus on the noise itself, which could have directly led to a higher score.

The test errors were analyzed in Section 3.3. The research method of this study incurred two types of test errors. For group A, the main test error occurred due to the microphone location inside the same vehicle, which led to an extreme error between approximately 3.5 and 5.4 dB(A). However, under the same annoyance score, the maximum difference of test SPL exceeded 20 dB(A). Due to the large differences in volunteers’ subjective evaluations of noise, the error caused by the microphone location difference in the vehicle was acceptable in this study. For group B, the main test error occurred due to the noise difference between vehicles. The maximum range reached approximately 8.5 dB(A), which led to low *adj*-*R*^2^ values in group B. Therefore, if the conditions allow it, the method used for group A should be used as far as possible. If the conditions do not allow this, however, some samples of group B could be mixed with those of group A. However, the samples of group B cannot be used alone to obtain the exposure-response relationship, due to the large error results caused by the noise difference between different vehicles.

### 4.2. Evaluation Indicator for Interior Noise

Equivalent SPL, which was applied in this study, is widely used to evaluate interior noise. The maximum A-weighted SPL (*L*_Amax_) and sound exposure level (SEL) can also be used to describe noise. *L*_Amax_ is usually used to describe the maximum value of SPL when a train is running between two stations. If the *L*_Amax_ increases instantaneously and lasts for a very short time, the responses of different passengers will be different. Some volunteers will remember the maximum value and use it as a basis for rating a high annoyance score, but some volunteers will consider its short duration, and will still give the annoyance score according to the average noise level between two stations. Due to the randomness of *L*_Amax_ and the different subjective cognitions of passengers regarding the maximum value, it was not suitable as an evaluation index in this study.

SEL is the total A-weighted sound energy over a certain time interval; it is expressed as the equivalent of time over 1 s, and is defined as:

(5) $$\mathrm{SEL}=10\mathrm{lg}\left\{\frac{1}{t_{0}}\int_{t_{1}}^{t_{2}}{\left[\frac{P_{A}\left(t\right)}{P_{0}}\right]}^{2}dt\right\}$$

where *t*_0_ is a reference time, usually with the value of 1 s.

**Figure 14:**
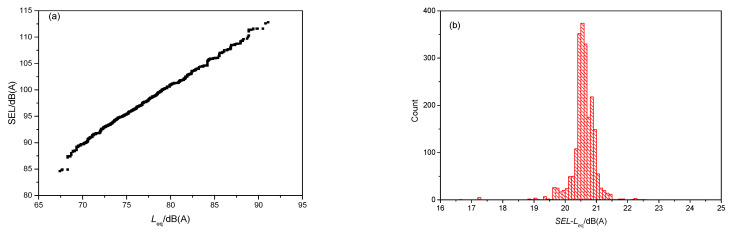
Relationship between SEL and equivalent SPL: (**a**) SEL vs. *L*_eq_ and (**b**) statistical result of the difference between SEL and *L*_eq_ for all test samples.

Theoretically, SEL responds to the energy of the whole test time, which is more related to the subjective annoyance of passengers. In fact, however, due to the similar distances between metro stations in urban areas, the train running times between any two stations are relatively similar. Accordingly, the equivalent SPL and the SEL exhibit a high correlation. Figure 14 illustrates the statistical relationship between SEL and equivalent SPL for all test samples. Figure 14a shows that there was a linear relationship between these two indicators. Figure 14b illustrates the statistical result of the difference between SEL and *L*_eq_; there was an average difference of 20.55 dB(A). This means that the effect of evaluating noise annoyance by SEL was very similar to that achieved by using SPL. However, if the samples from urban rail transit and suburban rail transit were both to be collected, there would be a significant difference in train running times between different stations. In this case, the SEL may be the better evaluation index. However, the samples collected in this study seldom involved suburban rail transit.

Another problem that needs to be addressed is that when using equivalent SPL, it is difficult to consider the influences of different noise frequencies on passengers’ annoyance fully. Volunteers’ subjective feedback suggested that high-frequency interior noise (e.g., >1000 Hz) caused by rail wear and other factors can probably cause high annoyance. However, low-frequency noise caused by laying floating slab tracks and other factors cannot easily annoy passengers, even though it has a high total SPL. However, despite the disadvantages mentioned above, the statistical study in this paper exhibits that the equivalent SPL is still a good index for evaluating passengers’ annoyance caused by interior noise.

### 4.3. Interior Noise Limits

At present, there is no interior noise standard considering passengers’ annoyance in China. In the Chinese national standard GB 14892 [53], ‘Noise limit and measurement for train of urban rail transit’, there are limits for the design, manufacture, and inspection of metro vehicles, but not for passenger comfort. According to this limit, there are no passengers on the train during the noise test. For an underground metro line, the suggested maximum allowable equivalent SPLs are 80 dB(A) and 83 dB(A) for the cab and passenger cars, respectively.

Unlike in the test environment in the above standard, in a regular operating metro train, there is not only broadcasting sound but also passenger conversation. This makes the interior noise during regular operation louder than that during the inspection. Therefore, based on the above specification limits, Table 4 analyses show the annoyance percentage and annoyance score corresponding to 80–85 dB(A). The exposure-response relationship used here was the fitting result obtained for group A. When the interior noise exceeded 80 dB(A), the percentages of annoyance and slight annoyance were extremely high. As discussed in Section 4.1, this was probably induced by the method adopted in this study. Therefore, the fitting results of high annoyance should be considered in greater detail. When the equivalent SPL increased from 80 dB(A) to 85 dB(A), the percentage of highly annoyed volunteers increased sharply from 31.6% to 79.5%. This reflects that passengers were extremely sensitive to changes in SPL values within this range. When the equivalent SPL reached 82 dB(A), more than half of the passengers may have felt ‘highly annoyed’. When the equivalent SPLs reached 84 and 85 dB(A), the corresponding average annoyance scores were 8.0 and 8.3, which are the lower limits of ‘highly annoyed’.

According to the above analysis, from the perspective of passengers’ acoustic comfort, the equivalent SPL of interior noise should not exceed 84–85 dB(A) when a metro train runs between two stations. If the test noises in one metro vehicle were to exceed this recommended value in several sections, it would be necessary to pay attention to the vehicle condition, such as door tightness, wheel out-of-round level, etc. If the test noises in several vehicles were to exceed this recommended value during the same section, it would be necessary to pay attention to the track condition in this section, such as abnormal rail corrugation.

Analysis of all noise test samples revealed that 6.7 and 5.1% of samples exceeded 84 and 85 dB(A), respectively. Although these proportions are not high, interior noise is a problem for the Beijing metro, which is a huge system with a total mileage of nearly 700 km; it still needs to be addressed and solved.

**Figure 15:**
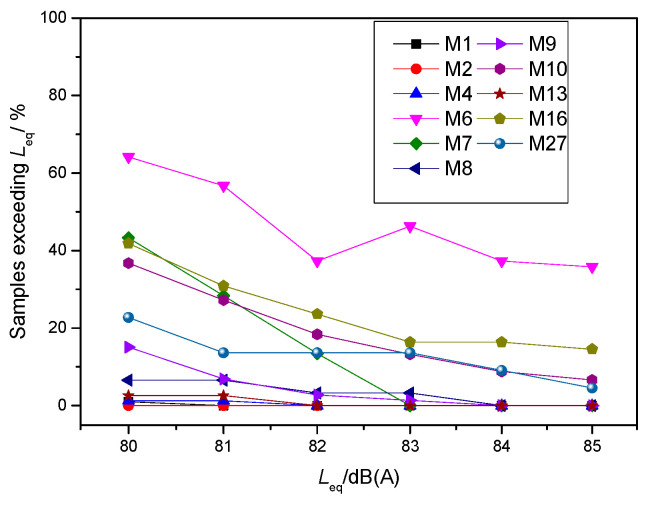
Proportions of samples exceeding different equivalent SPLs.

Figure 15 illustrates the proportions of test samples from different metro lines that exceeded the corresponding equivalent SPL. Regarding equivalent occurrences of SPL exceeding 84 dB(A), only for samples from M6 and M16 did this happen more frequently than 20%. In addition, the values of M6 were extremely abnormal, which is consistent with the fact that passengers complained about the interior noise of this line.

## 5. Conclusions

A research strategy was proposed to investigate the relationship between the metro interior noise and passengers’ annoyance based on interior noise tests and questionnaires completed by volunteers. The feedback from the questionnaire concerned eleven metro lines in Beijing. A total of 118 volunteers were divided into two groups: A and B. The volunteers of group A took the same metro train as the testers, whereas those of group B took different trains. The metro interior noises were tested, and a total of 2080 samples were collected and analyzed. The results indicated that:

(1) There was a significant positive correlation between the average subjective annoyance and the average equivalent SPL.
(2) The logistic function could accurately fit the relationship between interior noise and annoyance score/annoyance percentage.
(3) The fitting result was best for samples from group A, and was worst for samples from group B. For the mixed samples of both groups, the fitting result was greatly affected by the contribution of group A.
(4) To provide a comfortable acoustic environment, the metro interior noise should not exceed 84–85 dB(A).

Finally, it should be highlighted that the results of this study and the obtained exposure-response relationship were only based on subjective evaluations on the noise of eleven metro lines in the Beijing urban area from 118 volunteers. There are two limitations of this study: one is the number of participants, which brings uncertainties in results; another is the results from group B, in which the separation of subjective annoyance scoring and noise testing induced an unacceptable fitting results. To analyze the influence of noise on passengers comprehensively, more noise test data are suggested to collect according to the model of group A, and more volunteers with different ages should be involved in the future, which would help to improve the research results.

## Figures and Tables

**Table 1:** Contents of questionnaire.

Part 1: Basic information			
Sex		Age	
Current occupation		Metro line taken	
Part 2: Subjective rating of noise annoyance			
No.	Leaving from station	Approaching station	Subjective rating (from 0 to 10)
Section 1	*Station A*	*Station B*	
Section 2	*Station B*	*Station C*	
Section 3	*Station C*	*Station D*	
Section 4	*Station D*	*Station E*	
…	…	…	
Part 3: Perception description			
When you rated a high subjective score, what were the main effects of the interior noise on you?			

**Table 2:** Fitting parameters and fitting evaluation for the relationship between annoyance score and SPL.

Logistic Approximation: $\psi =\frac{10}{1+e^{-k\left(L_{\mathrm{eq}}-L_{c}\right)}}$	*k*	*L*_c_	*adj*-*R*^2^
For groups A and B	0.1452	77.0530	0.9512
For group A only	0.1795	76.2214	0.9789
For group B only	0.1003	78.3627	0.7523

**Table 3:** Fitting parameters and evaluations for the relationship between annoyance percentage and SPL.

Logistic Approximation: $\psi \%=\frac{100}{1+e^{-k\left(L_{\mathrm{eq}}-L_{c}\right)}}$		*k*	*L*_c_	*adj*-*R*^2^
For groups A and B	HA%	0.3919	83.0142	0.9746
	A%	0.2919	76.7671	0.9594
	LA%	0.3471	70.1986	0.9440
For group A only	HA%	0.4250	81.8174	0.9869
	A%	0.3571	75.8275	0.9534
	LA%	0.3588	70.3003	0.9716
For group B only	HA%	0.2561	86.1212	0.6188
	A%	0.2460	78.0151	0.8743
	LA%	0.3336	70.1809	0.8411

**Table 4:** Annoyance percentage and annoyance score corresponding to 80–85 dB(A) according to the fitting results of group A.

*L*_eq_/dB(A)	HA%	A%	LA%	Annoyance Score
80	31.6%	81.6%	97.0%	6.6
81	41.4%	86.4%	97.9%	7.0
82	51.9%	90.1%	98.5%	7.4
83	62.3%	92.3%	99.0%	7.7
84	71.7%	94.9%	99.3%	8.0
85	79.5%	96.4%	99.5%	8.3

## Data Availability

Not applicable.
